# Physical Activity, Sedentary Time, and Cardiovascular Disease Biomarkers at Age 60 to 64 Years

**DOI:** 10.1161/JAHA.117.007459

**Published:** 2018-08-08

**Authors:** Ahmed Elhakeem, Rachel Cooper, Peter Whincup, Soren Brage, Diana Kuh, Rebecca Hardy

**Affiliations:** ^1^ MRC Integrative Epidemiology Unit Population Health Sciences Bristol Medical School University of Bristol United Kingdom; ^2^ MRC Unit for Lifelong Health and Ageing at UCL London United Kingdom; ^3^ Population Health Research Institute St George's University of London United Kingdom; ^4^ MRC Epidemiology Unit University of Cambridge United Kingdom

**Keywords:** accelerometers, aging, cardiac biomarkers, heart rate, older adults, physical exercise, sedentary time, Epidemiology, Risk Factors, Exercise, Cardiovascular Disease, Primary Prevention

## Abstract

**Background:**

We examined associations of objectively measured physical activity (PA) and sedentary time with cardiovascular disease biomarkers at age 60 to 64 years. This included investigation of sex differences and the extent to which associations may be mediated by adiposity.

**Methods and Results:**

Participants were 795 men and 827 women aged 60 to 64 years from the Medical Research Council National Survey of Health and Development. Combined heart rate and movement sensors worn for 5 consecutive days were used to derive overall PA energy expenditure, kJ/kg per day) and time spent sedentary (<1.5 metabolic equivalent of tasks), in light PA (1.5–3 metabolic equivalent of tasks) and moderate‐to‐vigorous intensity PA (>3 metabolic equivalent of tasks). Linear regression models were used to relate each PA parameter to inflammatory (C‐reactive protein, interleukin‐6), endothelial (tissue‐plasminogen activator, E‐selectin) and adipokine (leptin, adiponectin) markers extracted from fasting blood samples. Greater time in light PA and moderate‐to‐vigorous intensity PA and less sedentary time were associated with more favorable biomarker levels. For C‐reactive protein, interleukin‐6, and leptin, these differences were greater among women than men. For example, % differences (95% confidence intervals) in leptin for men and women per SD increases in sedentary time: 7.9 (2.7, 13.0) and 20.6 (15.3, 25.8); moderate‐to‐vigorous intensity PA: −3.8 (−8.9, 12.7) and −17.7 (−23.1, −12.4), moderate‐to‐vigorous intensity PA: −12.9 (−17.9, −8.0) and −18.3 (−23.4, −13.1). Fat mass mediated a greater proportion of these associations in women than men.

**Conclusions:**

Greater light PA and moderate‐to‐vigorous intensity PA and less sedentary time in early old age were associated with more favorable cardiovascular biomarker profiles. Fat mass partially mediated these associations but more strongly in women than men, which explained sex differences.

**Clinical Perspective**

**What Is New?**

- We examined how time spent sedentary and in different activity intensities (as well as cardiorespiratory fitness) relates to inflammatory, endothelial, and adipokine markers in 60‐ to 64‐year‐old men and women.
- Less sedentary time and greater time in low and moderate‐to‐vigorous intensity activity were associated with favorable biomarker profiles.
- Fat mass partly explained associations and to a greater extent in women.
- Differences in interleukin‐6, tissue plasminogen activator, and leptin by sedentary time and light activity were independent of higher intensity activity.
- Cardiorespiratory fitness was associated with biomarkers, but this was largely mediated by fat mass.
- Physical activity volume was related to biomarkers independently of fitness.

**What Are the Clinical Implications?**

- Sedentary and inactive older adults should be supported to replace time spent sedentary with any intensity of physical activity.
- Activities that can reduce fat mass may be most beneficial for cardiovascular health.

## Introduction

Physical inactivity is a well‐known risk factor for cardiovascular disease (CVD)^1^, ^2^ and premature mortality from CVD.^2^, ^3^, ^4^ One explanation for these findings is thought to be because of beneficial effects of physical activity (PA) on endothelial function and the relatively less well‐studied biomarkers of atherosclerosis.^5^, ^6^, ^7^, ^8^, ^9^, ^10^, ^11^, ^12^ The anti‐inflammatory effects of PA have been well documented with consistent findings of decreases in pro‐inflammatory markers C‐reactive protein (CRP) and interleukin‐6 (IL‐6) following brief exercise interventions,^8^, ^9^ in addition to decreases in leptin and increases in adiponectin levels with exercise‐induced fat loss.^5^ PA also improves endothelial function in terms of its ability to release the protein tissue‐plasminogen activator (t‐PA), but the influence of PA on the cell adhesion molecule E‐selectin is uncertain.^6^ A better understanding of how PA relates to these markers may provide insight into underlying pathways leading to the development of atherosclerosis.^13^

Few epidemiological studies have examined relations between PA intensity and these CVD biomarkers, with the majority focusing on moderate‐to‐vigorous‐intensity PA (MVPA). Light‐intensity PA (LPA) makes up a growing proportion of the amount of time spent in PA at older age,^14^, ^15^ meaning it is also important to identify whether LPA in later life provides cardiovascular benefit or whether higher PA intensities are needed. This is challenged by reliance on poor estimates of PA intensity (especially lower‐intensity PA) obtained from self‐reported data; thus, studies where PA is objectively assessed are needed. Furthermore, sedentary behavior, where energy expenditure is minimal and prolonged lying or sitting is the dominant posture, has also been related to an increased CVD risk independently of PA,^16^, ^17^ though little is known on how time spent sedentary relates to CVD biomarkers at older age.

Importantly, whether associations between PA parameters and these CVD biomarkers vary between men and women is unknown, since the few existing studies either only included men^18^ or did not report tests for sex differences,^19^, ^20^ despite hypothesized sex differences in these associations.^21^ Moreover, higher adiposity, which is an established CVD risk factor,^22^ has also been related to an adverse biomarker profile,^23^ and might help explain associations between PA and CVD biomarkers^24^; however, to what extent adiposity mediates these associations is unclear, especially as PA and adiposity associations are still widely debated.^25^, ^26^ Additionally, among older adults, women tend to have a higher fat mass for a given body mass index than men,^27^ meaning it is important to study the mediating role of more direct measures of adiposity. Targeting adiposity could be included within all‐inclusive PA interventions and likewise, a better understanding of underlying sex‐differences would also inform intervention design.

Therefore, the aim of this study was to examine how overall PA volume, time spent sedentary and at lower and higher PA intensities relate to CVD biomarkers in early old age. Specifically, we address important gaps in the existing literature by testing whether findings differed by sex, whether LPA and sedentary time were related to biomarkers independently of MVPA, and the extent to which adiposity mediates associations and explains sex‐differences. We hypothesized that greater time spent in MVPA and LPA and less sedentary time would be associated with more favorable levels of CVD biomarkers, and that fat mass would partly mediate these associations.

## Methods

### Study Population

Data used in this publication are available to bona fide researchers upon request to the MRC National Survey of Health and Development (NSHD) Data Sharing Committee via a standard application procedure. Further details can be found on the study website and data set doi's (https://www.nshd.mrc.ac.uk/).^28^ The MRC NSHD is a socially stratified British cohort of 5362 males and females followed up at regular intervals since birth during 1 week in March 1946.^29^, ^30^ Between 2006 and 2010 (at age 60–64), a total of 2856 eligible study members (those still alive and with a known address in England, Scotland, or Wales) were invited for an assessment at 1 of 6 clinical research facilities, or to be visited by a research nurse at home. Invitations were not sent to those who had died (n=778), were living abroad (n=570), had previously withdrawn from the study (n=594), or been lost to follow‐up (n=564). Of those invited, 2229 (78%) were assessed: 1690 attended a clinical research facility and the remaining 539 were seen at home.^31^

Ethical approval for the study at age 60 to 64 years was obtained from the Greater Manchester Local Research Ethics Committee and the Scotland A Research Ethics Committee. Written, informed consent was obtained from study members for each component of the data collection.

### Cardiovascular Disease Biomarkers

Overnight fasting blood samples were taken during the clinical assessment and initially processed at the clinical research laboratories. Aliquots were frozen and stored before being transferred to the MRC Human Nutrition Research laboratory in Cambridge where analyses of inflammatory marker CRP was processed according to standardized protocols. Analyses of adipokines (leptin and adiponectin), endothelial markers (E‐selectin and t‐PA), and inflammatory marker IL‐6 were undertaken by British Heart Foundation Research Centre in Glasgow. The method and commercial assay plus interassay coefficients of variation are given in Table S1.

### PA and Sedentary Time

At the end of their clinical assessment at age 60 to 64 years, study participants were invited to wear a combined movement (from acceleration) and heart rate monitor (Actiheart, CamNtech, Ltd, Papworth, United Kingdom) attached to their chest for 5 consecutive days.^32^ Heart rate and movement data were recorded in 30‐s epochs to derive measures of free‐living PA. Heart rate data were preprocessed^33^ and then individually calibrated using an 8‐minute step test to account for between‐individual differences in the relationship between PA intensity and heart rate^34^; group calibration was used where individual calibration was not carried out, adjusted only for sleeping heart rate, age, sex, and β‐blocker use.^34^ Total PA energy expenditure (PAEE) (in kJ/kg per day) was derived using a branched equation framework^35^ as recently validated in a UK population^36^ from which time spent at different PA intensities was summarized; these were based on metabolic equivalent of tasks (METs) as time spent sedentary (<1.5 METs) and in LPA (1.5–3 METs) and MVPA (>3 METs), using 1 standard MET (20.35 J/ mL O_2_×3.5 mL O_2_/ min per kg) as resting metabolic rate.

This standard definition of 1 MET, which summarized intensity regardless of body composition, was used so as to avoid overestimation of PA intensity with higher body mass index. PA parameters were adjusted for wear time and diurnal information bias to allow for variation between individuals in wear time at different times of the day when levels of PA may be expected to vary. Following visual inspection of heart rate and movement traces, participants were excluded if acceleration signals were corrupted or where valid heart rate measurements were not available (n=55). Those with <48 hours of wear time (n=24) were excluded to ensure PA data were an accurate reflection of normal PA. All PA parameters were sex‐standardized to mean=0 and SD=1. In addition, in the subsample with sufficient step test data (≥4 minutes), we derived cardiorespiratory fitness by extrapolating the submaximal relationship between heart rate and energy expenditure to age‐predicted maximal heart rate; the latter was adjusted downwards by 20 beats per minute for individuals on β‐blockers to reflect their lower maximal heart rate.^37^ The estimate was expressed as maximal oxygen uptake (VO_2_max; mL O_2_/min per kg).

### Covariates

Socioeconomic position, smoking history, long‐term illness, health problem or disability, blood pressure, diabetes mellitus, CVD, and medication use were selected a priori for inclusion in analyses. Educational level and occupational class were used as indicators of socioeconomic position. Education was based on highest level attained by age 26 and categorized as none; vocational/O‐levels or their equivalents; A‐levels or their equivalents; and degree or higher. Occupational class was based on the Registrar General's social classification at age 53 (or at younger ages if missing) and categorized as professional and intermediate (I&II); skilled nonmanual (IIINM); skilled manual (IIIM); and semiskilled and unskilled manual (IV&V). Smoking history up to age 60 to 64 years was grouped as never, former, or current. At age 60 to 64 years, participants reported whether they had any long‐term illness, health problems or disability that limits their activity, and self‐reported doctor diagnoses of diabetes mellitus, stroke, angina, and myocardial infarction. Also, at age 60 to 64 years, blood pressure was assessed by nurses using an Omron device, and participants reported their use of antihypertensive medication, β‐blockers, nitrates, and lipid‐lowering drugs. Age at measurement was recorded.

We hypothesized that body size and specifically adiposity may mediate associations between PA parameters and CVD biomarkers. As a result, fat mass index (whole body fat mass excluding head (kg)/height (m)^1.2^, standardized to mean=0 and SD=1) obtained from QDR 4500 Discovery dual‐energy X‐ray absorptiometry scanner (Hologic Inc, Bedford, MA) among the subsample of participants who attended a clinical research facility at age 60 to 64 years was included in analyses.

### Statistical Analysis

All biomarkers (CRP, IL‐6, E‐selectin, t‐PA, leptin, and adiponectin) were positively skewed and therefore transformed using the natural logarithm. All analyses were carried out separately in men and women (with formal tests of sex interactions in associations of each PA parameter and each biomarker tested in models with men and women combined). Evidence of nonlinearity was examined by adding a quadratic term for each PA parameter measure, with quadratic models retained if evidence of nonlinearity was found.

Initial models were adjusted for age and subsequent models were further adjusted for covariates (socioeconomic position, smoking history, long‐term illness, health problem or disability, blood pressure, medication use, and CVD). To examine the influence of adiposity on any sex differences found, we fitted third models with additional adjustment for fat mass index (sex‐stratified models and sex‐combined models with interaction terms for PA parameter‐by‐sex). We also used these third models to explore mediation via adiposity, by comparing the degree of attenuation in estimates before and after adjustment for fat mass index. To examine whether sedentary time and LPA were associated with biomarkers independently of MVPA, we fitted additional models with mutual adjustment for (1) sedentary time and MVPA, and (2) LPA and MVPA, similar to a recent study examining independence of these associations in older men.^18^ This approach was chosen since a high degree of multicollinearity precluded simultaneous adjustment for all 3 PA measures.

To minimize the potential for bias because of missing data in these regression analyses, we used multiple imputation by chained equations^38^ to impute missing data for all covariates in participants with complete data on PA parameters and at least 1 biomarker (n=up to 387 men and 312 women in total; education: n=86, occupational class: n=8, smoking history: n=136, long‐term illness, health problem, or disability: n=4, blood pressure: n=7, diabetes mellitus: n=132, angina and/or myocardial infarction: n=175, stroke: n=133, medication use: n=0, and fat mass index: n=398). Imputation models were fitted separately for each biomarker and run using 20 multiply imputed data sets that were combined using Rubin's combination rules. Findings from multiple imputation and complete‐case analyses were similar so the former are presented. All results were presented as percentage difference in biomarker levels since biomarkers are logged.^39^

In additional analyses, we used structural equation modeling to quantify the degree of mediation (and confirm partial mediation) related to our hypothesized mediation model by estimating direct and indirect (through fat mass) paths between each PA parameter and each biomarker. These models were estimated separately in men and women after adjustment for all covariates using full information maximum likelihood to deal with missing data. Finally, we performed an additional analysis (after multiple imputation of missing covariates) to examine how cardiorespiratory fitness relates to these biomarkers and the role of fat mass in explaining these associations. We also performed a final model with both VO_2_max and PAEE to examine whether they were independently related to biomarkers. All analyses were carried out in STATA 14 (StataCorp, College Station, TX).

### Sensitivity Analyses

To examine whether associations differed substantially in those with clinically manifest CVD or diabetes mellitus, we refitted all models after excluding those with CVD (stroke, angina, and myocardial infarction) and diabetes mellitus diagnoses up to age 60 to 64 years (n=up to 201 men and 162 women). Additionally, we re‐examined associations with CRP after excluding levels indicative of acute infection (>10 mg/L) (n=47 men and 50 women). We also repeated analyses after excluding those on β‐blockers (n=174), as this may affect quantification of PAEE and fitness from heart rate data. Lastly, analyses were repeated using average trunk acceleration (m/s^2^) as a measure of overall PA instead of PAEE.^27^

## Results

### Descriptive Statistics

**Figure 1:**
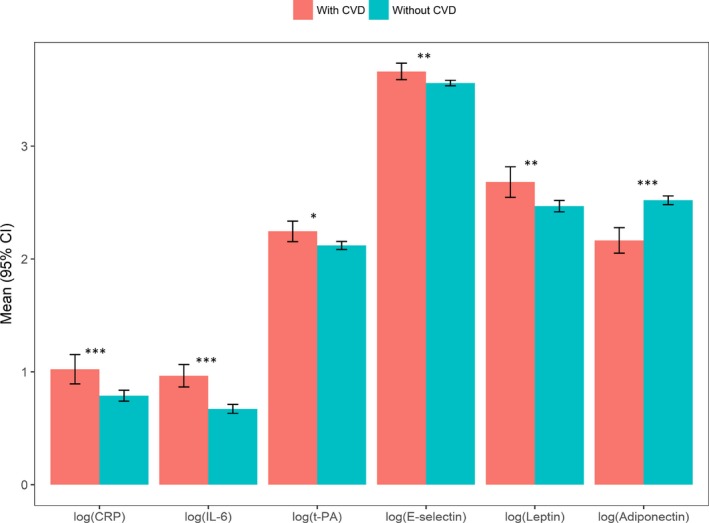
Mean biomarkers in those without and with cardiovascular disease (CVD) diagnoses. Median biomarker values in those without and with CVD diagnoses, respectively, were: CRP=2 and 2.5 mg/L, IL‐6=1.8 and 2.4 (pg/mL), t‐PA=9.0 and 10.2 (ng/mL), E‐selectin=36.5 and 39.1 (ng/mL), leptin=11.8 and 13.9 (ng/mL), and adiponectin=12.8 and 8.3 (μg/mL). CVD diagnoses include diabetes mellitus, stroke, angina, and myocardial infarction. *P≤0.05, **P≤0.01, ***P≤0.001. CRP indicates C‐reactive protein; IL‐6, interleukin‐6; t‐PA, tissue plasminogen activator.

Of the 2229 participants with a clinic or home visit at age 60 to 64 years, 2065 (92.6%) had data on ≥1 biomarker and, of these, 1622 (51.0% female) also had data on PA and sedentary time (Table 1). Women had higher levels of adiponectin and leptin than men, while men had higher levels of E‐selectin and t‐PA (Table 1). Men spent greater time in MVPA than women (Table 1). Participants without CVD had more favorable levels of biomarkers than those with CVD (Figure 1). Those without CVD had higher median PA and lower sedentary time than those with CVD (PAEE: 35.0 versus 30.0 kJ/kg per day; MVPA: 0.58 versus 0.40 h/d; LPA: 5.4 versus 4.7 h/d; sedentary time: 17.8 versus 18.7 h/d). Mean VO_2_max was higher in men than women (31.4 versus 28.5 mL O_2_/min per kg) and in those without CVD (30.1 versus 29.3 mL O_2_/min per kg).

**Table 1:** Characteristics of Participants From the Medical Research Council National Survey of Health and Development With Data on Monitored Physical Activity and Sedentary Time and at Least 1 Biomarker at Age 60 to 64 Years

	Men (n=795)	Women (n=827)	*P* Sex Difference
Cardiovascular disease biomarkers (geometric mean [95% CI])			
Inflammatory markers			
C‐reactive protein (mg/L)	2.2 (2.1, 2.3)	2.4 (2.2, 2.5)	0.700
IL‐6 (pg/mL)	2.1 (2.0, 2.2)	2.0 (1.9, 2.1)	0.108
Endothelial markers			
Tissue‐plasminogen activator (ng/mL)	8.9 (8.5, 9.3)	8.0 (7.7, 8.4)	<0.001
E‐selectin (ng/mL)	36.2 (35.1, 37.4)	34.1 (33.1, 35.2)	0.004
Adipokines			
Leptin (ng/mL)	7.3 (6.9, 7.7)	20.0 (19.0, 21.2)	<0.001
Adiponectin (μg/mL)	8.8 (8.3, 9.2)	15.9 (15.3, 16.6)	<0.001
Physical activity variables (median [25th, 75th percentiles])			
PAEE (kJ/kg per day)	35.7 (26.9, 47.5)	33.5 (25.2, 41.8)	<0.001
Sedentary time (<1.5 METs) (h/d)	17.9 (16.2, 19.4)	18.0 (16.6, 19.4)	0.317
Light‐intensity activity (1.5–3 METs) (h/d)	5.2 (4.0, 6.5)	5.4 (4.3, 6.7)	0.042
MVPA (>3 METs) (h/d)	0.7 (0.3, 1.2)	0.4 (0.2, 0.8)	<0.001
Covariates			
Educational level (N [(%)])			
None	238 (31.5)	237 (30.0)	<0.001
Vocational/O‐level GCSEs	153 (20.3)	285 (36.1)	
A‐level GCSEs	236 (31.3)	220 (27.9)	
Degree or equivalent	128 (17.0)	47 (6.0)	
Occupational class (N [%])			
I&II	444 (55.9)	331 (40.0)	<0.001
IIINM	85 (10.7)	289 (35.0)	
IIIM	192 (24.2)	65 (7.9)	
IV&V	74 (9.3)	142 (17.2)	
Smoking history (N [%])			
Current smoker	71 (9.8)	89 (11.6)	0.013
Ex‐smoker	440 (60.4)	405 (52.9)	
Never smoked	217 (29.8)	272 (35.5)	
Long‐term illness, health problem, or disability (N [%])			
No	624 (78.3)	619 (74.7)	0.085
Yes	173 (21.7)	210 (25.3)	
Ever diagnosed with diabetes mellitus (N [%])			
No	672 (92.6)	730 (94.6)	0.115
Yes	54 (7.4)	42 (5.4)	
Ever diagnosed with angina or MI (N [%])			
No	650 (91.4)	718 (96.6)	<0.001
Yes	61 (8.6)	25 (3.4)	
Ever diagnosed with stroke (N [%])			
No	712 (97.9)	759 (98.6)	0.347
Yes	15 (2.1)	11 (1.4)	
Use of β‐blockers (N [%])			
No	706 (88.4)	749 (90.1)	0.248
Yes	93 (11.6)	82 (9.9)	
Use of antihypertensive and/or heart failure drugs (N [%])			
No	611 (76.5)	686 (82.6)	0.002
Yes	188 (23.5)	145 (17.5)	
Use of nitrates (N [%])			
No	680 (85.1)	750 (90.3)	0.002
Yes	119 (14.9)	81 (9.8)	
Use of lipid‐lowering drugs (N [%])			
No	564 (70.6)	673 (81.0)	<0.001
Yes	235 (29.4)	158 (19.0)	
Diastolic blood pressure (mm Hg) (arithmetic mean (95% [CI])	79.4 (78.7, 80.1)	75.8 (75.2, 76.5)	<0.001
Fat mass index (kg/m^1.2^) (arithmetic mean [95% CI])	7.7 (7.5, 7.8)	11.0 (10.8, 11.3)	<0.001

Mean (SD) in physical activity (PA) parameters for men and women, respectively: PA energy expenditure=38.3 (15.8) and 34.5 (13.4) kJ/kg per day, moderate‐to‐vigorous‐intensity PA=0.9 (0.8) and 0.6 (0.6) h/d, light‐intensity PA=5.4 (1.8) and 17.9 (2.1) h/d, sedentary time=17.7 (2.2) and 17.9 (2.1) h/d. CI indicates confidence interval; GCSEs, General Certificate of Secondary Education; IL‐6, interleukin‐6; METs, metabolic equivalents of task; MI, myocardial infarction; MVPA, moderate‐to‐vigorous physical activity; PAEE, physical activity energy expenditure.

### PA and Inflammatory Markers (CRP and IL‐6)

**Figure 2:**
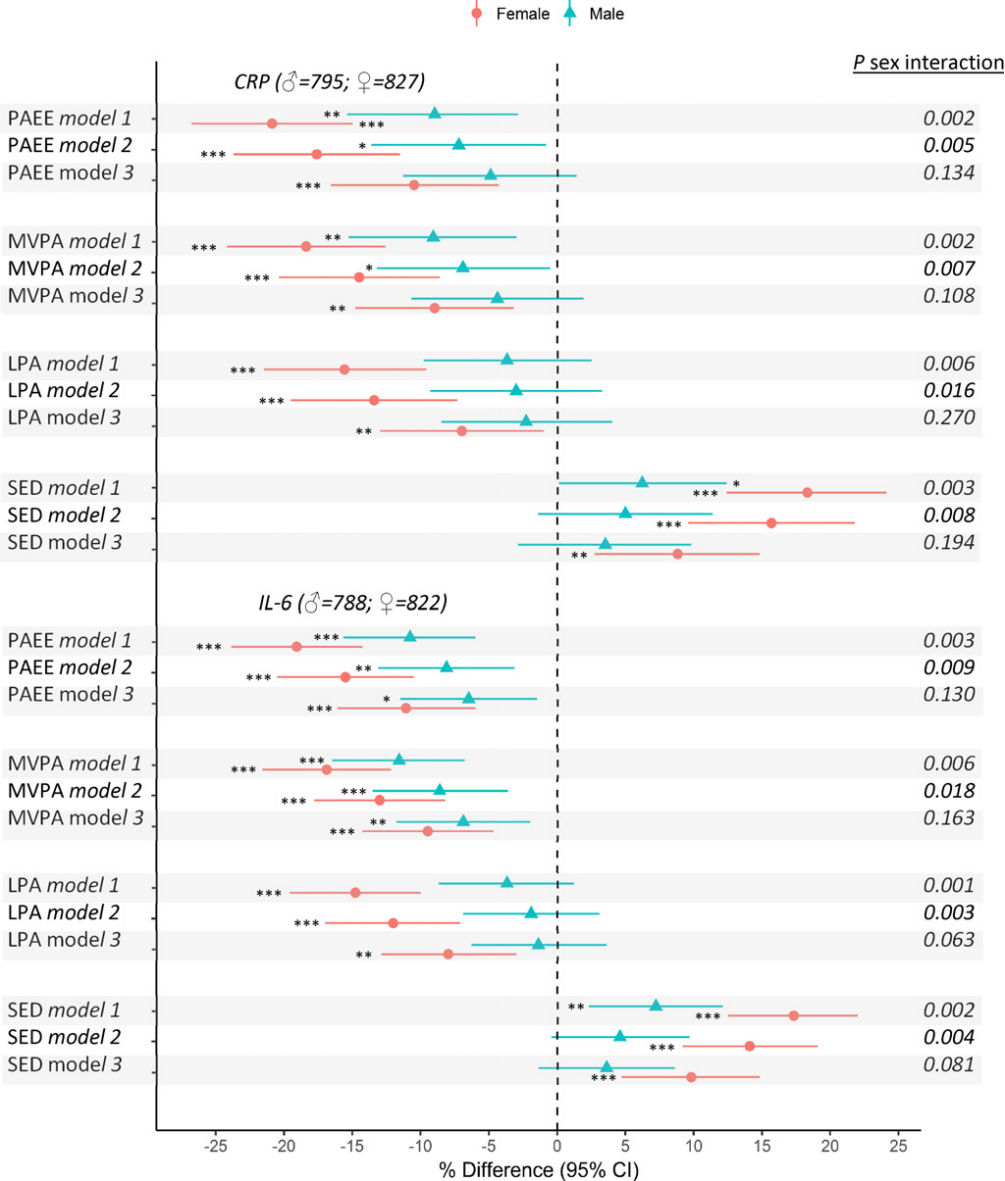
Mean percentage difference (95% confidence intervals) in inflammatory markers (CRP and IL‐6) per SD increases in time spent sedentary (SED), in light‐ (LPA) and moderate‐to‐vigorous‐intensity physical activity (MVPA), and overall physical activity energy expenditure (PAEE) at age 60 to 64 years. Model 1 adjusted for age. Model 2 adjusted for age, SEP, smoking history, long‐term illness, health problem or disability, blood pressure, CVD, and medication use. Model 3 as for model 2 plus adjustment for fat mass index. *P* for sex interaction from sex‐combined models. Sex interaction tests based on nonstandardized PA measures. *P≤0.05, **P≤0.01, ***P≤0.001. CI indicates confidence interval; CRP, C‐reactive protein; IL‐6, interleukin‐6.

**Figure 3:**
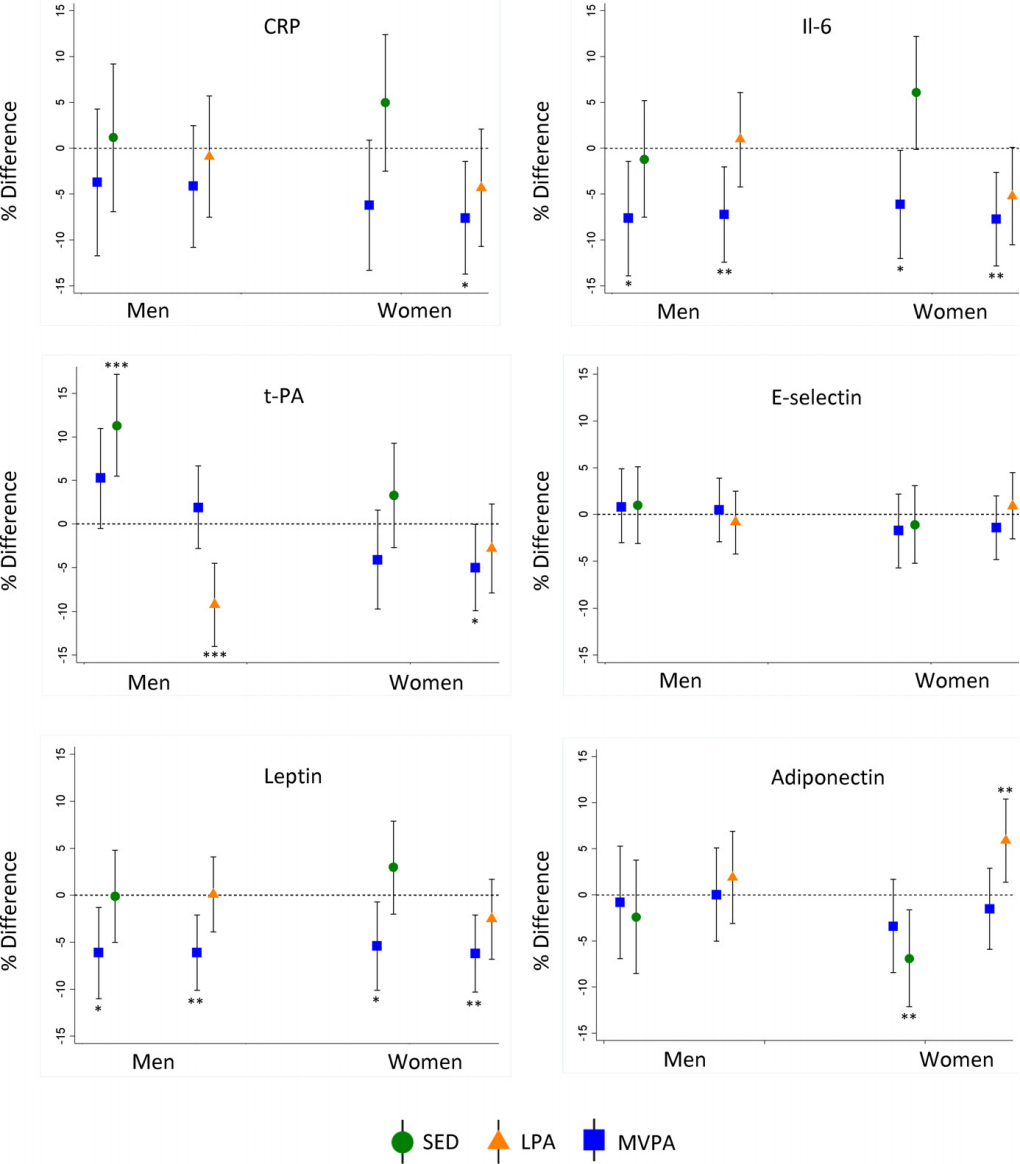
Mean percentage difference (95% confidence intervals) in biomarkers after mutual adjustment of sedentary time (SED) and light‐intensity physical activity (LPA) for moderate‐to‐vigorous‐intensity physical activity (MVPA). Models adjusted for covariates (age, SEP, smoking history, long‐term illness, health problem or disability, blood pressure, CVD, medication use, and fat mass index) plus MVPA (separately for LPA and SED). Estimates represent 1 SD increases in each PA parameter. *P≤0.05, **P≤0.01, ***P≤0.001. CRP indicates C‐reactive protein; CVD, cardiovascular disease; IL‐6, interleukin‐6; SEP, socioeconomic position; t‐PA, tissue plasminogen activator.

Greater sedentary time was associated with higher CRP and IL‐6 and associations were stronger in women than men and were maintained after adjustment for covariates (Figure 2). Accounting for fat mass largely reduced sex differences (Figure 2); fat mass partly mediated overall associations but to a greater extent for women (Figure 2, Table 2). Greater time spent in LPA and MVPA was related to lower CRP and IL‐6 in both sexes, but these associations were considerably stronger in women even after accounting for covariates (Figure 2). Further adjustment for fat mass attenuated sex‐differences (Figure 2) and only partially mediated associations, though with greater mediation in women than men (Figure 2, Table 2). Associations between PAEE and CRP and IL‐6 were similar to those observed for MVPA (Figure 2). The associations of sedentary time and LPA with IL‐6 (women) were maintained after adjustment for MVPA but associations with CRP largely attenuated (Figure 3). These adjustments had little influence on MVPA‐CRP/IL‐6 associations, with mutual adjustment for sedentary time resulting in a greater reduction in differences when compared with mutual adjustment for LPA (Figure 3).

**Table 2:** Percentage of the Association Between Each Physical Activity Parameter and Each Biomarker That Is Mediated by Fat Mass Index in Men and Women: Path Analysis

	PAEE	MVPA	LPA	SED
CRP				
Men	38.8	38.3	37.1	39.2
Women	50.6	49.8	57.7	54.5
IL‐6				
Men	22.7	20.9	35.0	26.7
Women	40.6	39.7	46.5	43.4
t‐PA				
Men	26.0	67.4	8.4	16.0
Women	52.3	49.3	57.7	54.3
E‐selectin				
Men	55.9	84.5	18.5	40.7
Women	76.0	77.8	84.1	82.1
Leptin				
Men	59.4	58.0	64.9	61.3
Women	67.7	63.3	71.7	69.1
Adiponectin				
Men	52.0	64.5	21.4	34.4
Women	54.3	92.7	38.6	43.8

Data show % of the total effects of each activity parameter that is mediated by fat mass index (indirect effect through fat mass/total effect). Adjusted for age, SEP, smoking history, long‐term illness, health problem or disability, blood pressure, cardiovascular disease, and medication use. CRP indicates C‐reactive protein; IL‐6, interleukin‐6; LPA, light‐intensity physical activity; MVPA, moderate‐to‐vigorous physical activity; PAEE, physical activity energy expenditure; SED, sedentary; SEP, socioeconomic position; t‐PA, tissue‐plasminogen activator.

### PA and Endothelial Markers (t‐PA and E‐Selectin)

**Figure 4:**
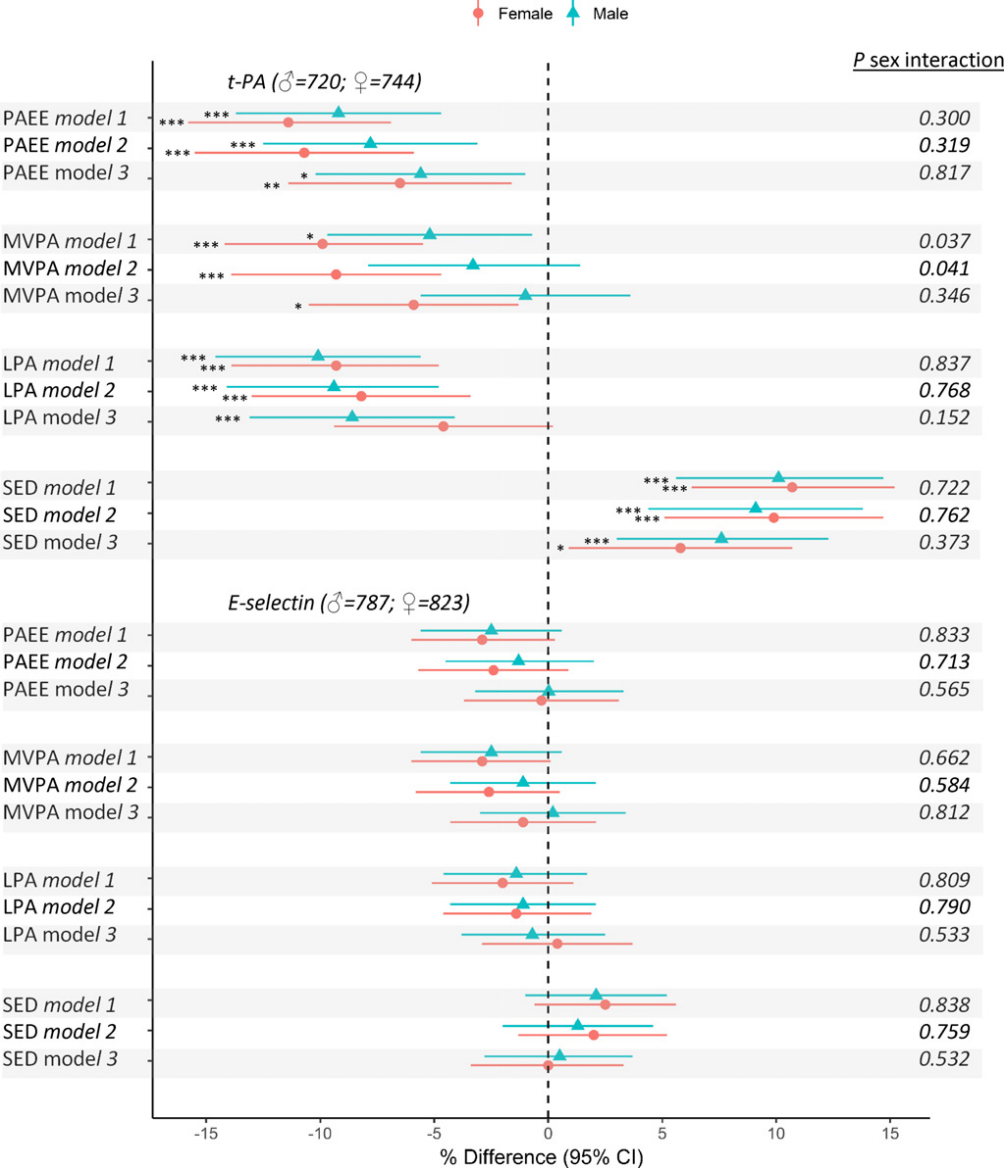
Mean percentage difference (95% confidence intervals) in endothelial markers (t‐PA and E‐selectin) per SD increases in time spent sedentary (SED), in light‐ (LPA) and moderate‐to‐vigorous intensity physical activity (MVPA), and overall physical activity energy expenditure (PAEE) at age 60 to 64 years. Model 1 adjusted for age. Model 2 adjusted for age, SEP smoking history, long‐term illness, health problem or disability, blood pressure, CVD, and medication use. Model 3 as for model 2 plus adjustment for fat mass index. *P* for sex interaction from sex‐combined models. Sex interaction tests based on nonstandardized PA measures. *P≤0.05, **P≤0.01, ***P≤0.001. CI indicates confidence interval; CVD, cardiovascular disease; SEP, socioeconomic position; t‐PA, tissue plasminogen activator.

Higher sedentary time was related to higher t‐PA in both men and women, including after accounting for covariates and fat mass (Figure 4). Higher LPA was related to lower t‐PA in both men and women, and this association was only slightly attenuated by adjustment for covariates and partially mediated by fat mass (Figure 4, Table 2). Greater time spent in MVPA was related to lower t‐PA in both sexes, but these associations were considerably stronger in women, even after accounting for covariates (Figure 4). Fat mass partially mediated these associations (Figure 4, Table 2). In both men and women, higher PAEE was associated with lower t‐PA both before and after adjustment for covariates and fat mass (Figure 4). Greater time in MVPA was weakly associated with lower E‐selectin but association was attenuated by adjustment for covariates whereas sedentary time, LPA, and PAEE were not associated with E‐selectin (Figure 4). Associations of sedentary time and LPA with t‐PA (men) persisted after adjustment for MVPA (Figure 3).

### PA and Adipokines (Leptin and Adiponectin)

**Figure 5:**
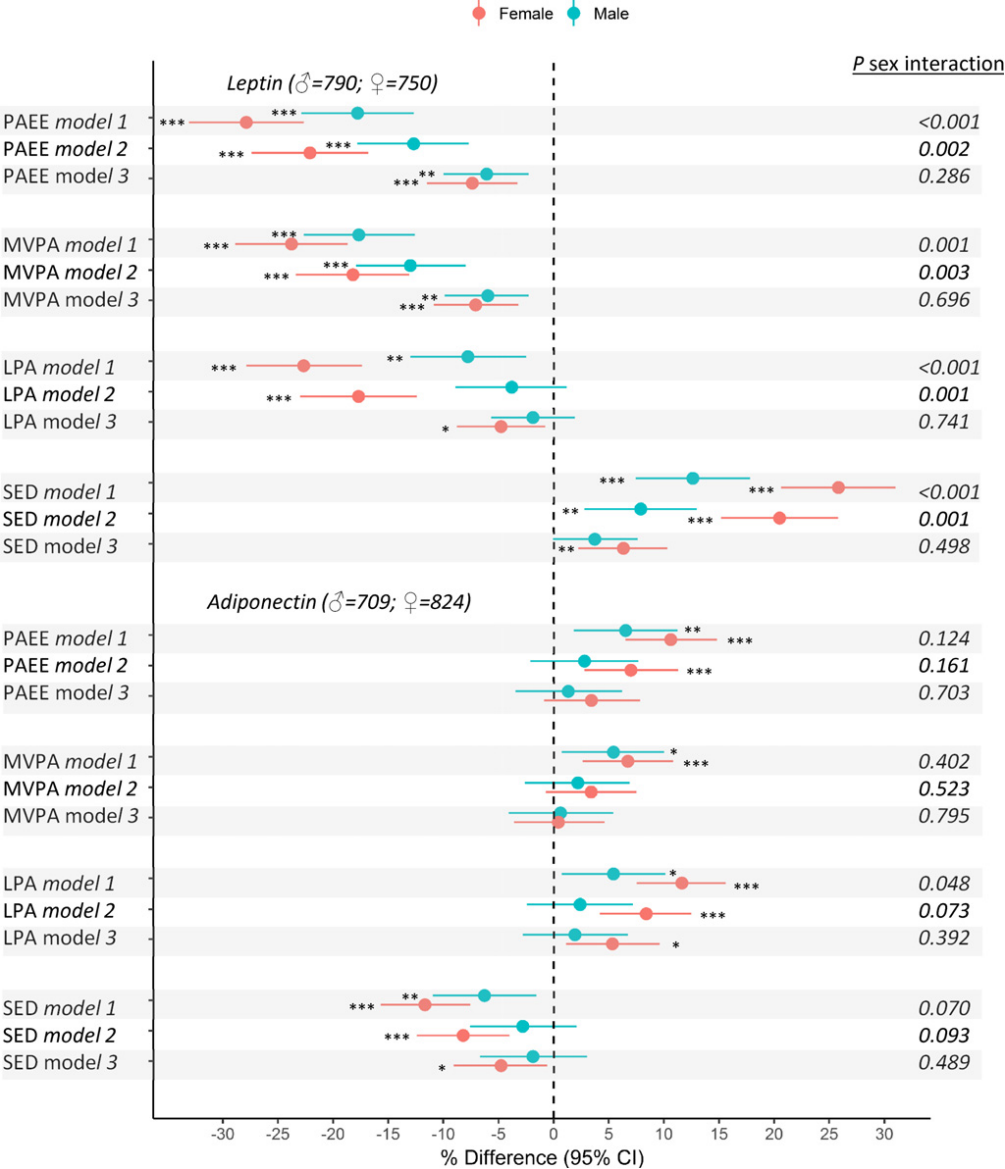
Mean percentage difference (95% confidence intervals) in adipokines (leptin and adiponectin) per SD increases in time spent sedentary (SED), in light‐ (LPA) and moderate‐to‐vigorous‐intensity physical activity (MVPA), and overall physical activity energy expenditure (PAEE) at age 60 to 64 years. Model 1 adjusted for age. Model 2 adjusted for age, SEP, smoking history, long‐term illness, health problem or disability, blood pressure, CVD, and medication use. Model 3 as for model 2 plus adjustment for fat mass index. *P* for sex interaction from sex‐combined models. Sex interaction tests based on nonstandardized PA measures. *P≤0.05, **P≤0.01, ***P≤0.001. CI indicates confidence interval; CVD, cardiovascular disease; SEP, socioeconomic position.

Greater sedentary time was associated with higher leptin and lower adiponectin including after adjustment for covariates, and differences were larger in women (Figure 5). Controlling for fat mass reduced these sex differences, but fat mass only partially mediated associations (Figure 5, Table 2). Greater LPA was associated with lower leptin and higher adiponectin in women but not men; associations persisted after adjustments and were partially mediated by fat mass (Figure 5, Table 2). MVPA was related to lower leptin and higher adiponectin in both sexes, but associations with leptin were stronger in women (Figure 5). Associations between MVPA and leptin were maintained, but those with adiponectin reduced after accounting for covariates (Figure 5). Adjustment for fat mass attenuated sex‐differences and partially mediated these associations (Figure 5, Table 2). Associations of PAEE with leptin and adiponectin were similar to those observed for LPA (Figure 5). Sedentary time and LPA remained associated with leptin and adiponectin (women) after adjustment for MVPA (Figure 3). These adjustments had little influence on MVPA‐leptin/adiponectin associations, with mutual adjustment for sedentary time resulting in a greater reduction in differences when compared with adjustment for LPA (Figure 3).

### Cardiorespiratory Fitness and CVD Biomarkers

**Figure 6:**
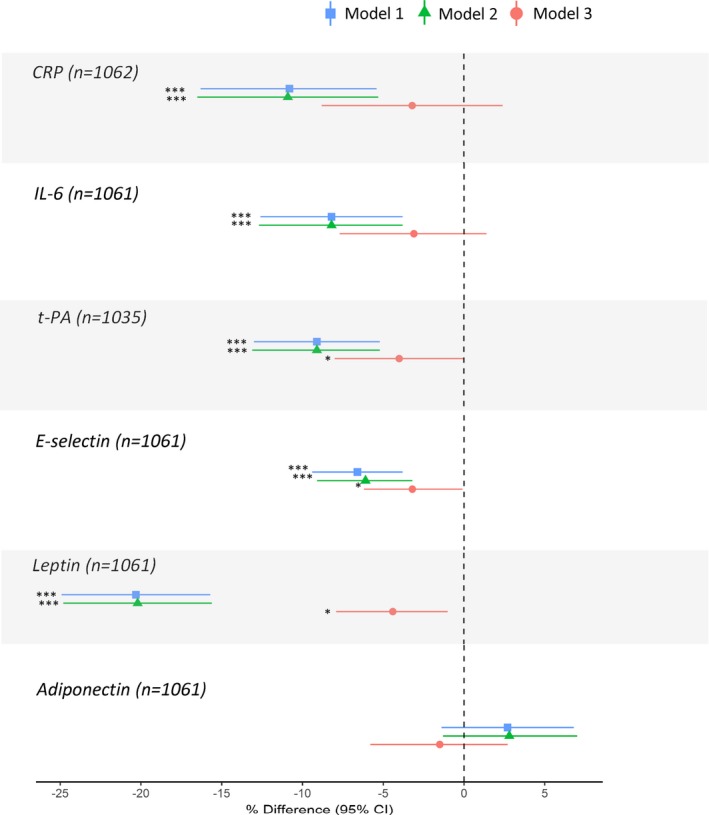
Mean percentage difference (95% confidence intervals) in biomarkers per SD increases in cardiorespiratory fitness (VO_2_max) at age 60 to 64 years. Model 1 adjusted for age and sex. Model 2 adjusted for age, sex, SEP, smoking history, long‐term illness, health problem or disability, blood pressure, CVD, and medication use. Model 3 as for model 2 plus adjustment for fat mass index. *P≤0.05, ***P≤0.001. CI indicates confidence interval; CRP, C‐reactive protein; CVD, cardiovascular disease; IL‐6, interleukin‐6; SEP, socioeconomic position; t‐PA, tissue plasminogen activator.

There was no evidence of sex‐interactions in the associations of VO_2_max estimates with CVD biomarkers (*P*>0.1 for all tests of interactions); therefore, results are presented for men and women combined (n=1035–1062). Higher VO_2_max was associated with better levels of all biomarkers except for the adipokine marker adiponectin, which showed no significant association; in contrast, the largest difference by VO_2_max was found for leptin (Figure 6). Adjustment for covariates had little influence on the associations found; however, in all cases, associations were largely mediated by fat mass (Figure 6). This was confirmed by path analysis, which showed that the proportion of associations mediated by fat mass were 89.7% for CRP, 85.5% for IL‐6, 80.0% for t‐PA, 68.3% for E‐selectin, and 82.4% for leptin. When VO_2_max and overall PA volume were included in the same model (after adjustment for all covariates and fat mass), SD differences in PAEE were inversely associated with CRP (−6.1%; −11.8 to −0.5), IL‐6 (−8.7%; −13.3 to −4.2), and leptin (−5.3%; −8.8 to −1.7), whereas VO_2_max was inversely associated with t‐PA (−3.6%; −7.8 to 0.7) and E‐selectin (−3.1%; −6.3 to 0.1).

### Sensitivity Analyses

Associations between PA parameters and biomarkers were similar after excluding participants with CVD and diabetes mellitus (Table S2). Associations with CRP were similar after excluding participants with levels >10 mg/L; for example, % changes in CRP per SD increase in time spent at MVPA was −2.0% (−6.8, 2.8) in men and −5.1% (−9.8, −0.6) in women (adjusted for covariates and fat mass index). Our findings were also similar after excluding participants on β‐blockers. When average trunk acceleration was used instead of PAEE, associations with E‐selectin and leptin were similar but associations with CRP, IL‐6 (men), t‐PA (women), and adiponectin (women) were weaker (not shown).

## Discussion

### Main Findings

We examined associations of time spent sedentary, in low‐ and moderate‐to‐vigorous‐intensity activity, and overall physical activity energy expenditure from individually calibrated combined heart rate and movement sensors with inflammatory, endothelial, and adipokine markers in 60‐ to 64‐year‐old men and women from a nationally representative British birth cohort. Except for E‐selectin, which largely showed no associations, less sedentary time and greater time spent in LPA and MVPA and higher PAEE were all associated with more favorable CVD biomarker profiles even after adjustment for a wide range of covariates. For CRP, IL‐6, leptin, and less so for adiponectin, associations were stronger among women than men (on both relative and absolute scales), but sex differences were largely explained by the greater fat mass of women for a given body mass index. Adiposity only partly mediated associations, and in general this mediation was greater in women than men. Except for t‐PA and leptin, differences in biomarker levels were greater for MVPA than sedentary time and LPA. Sedentary time and LPA were associated with IL‐6, t‐PA, and leptin independently of MVPA. In addition, we also found associations between higher cardiorespiratory fitness and favorable biomarker levels, which were largely mediated by adiposity.

### Comparison With Other Studies

This is one of the first studies to comprehensively investigate the associations of PA and sedentary time with a wide range of CVD biomarkers at older age in both men and women. Our findings are consistent with the few other studies that have investigated associations between some of these PA parameters and some of these CVD biomarkers. These include results from men aged in their 70s from the British Regional Heart Study showing that higher MVPA was associated with lower levels of IL‐6, CRP, and t‐PA, and that associations between sedentary time and IL‐6 or t‐PA were independent of MVPA.^18^ Our results also agree with findings from National Health and Nutrition Examination Survey showing that LPA and MVPA were related to better cardiometabolic biomarkers and that associations with triglycerides and blood pressure were stronger in women.^40^ Likewise, our results support other findings from National Health and Nutrition Examination Survey showing independent associations between higher sedentary time and more adverse levels of cardiometabolic biomarkers,^41^ and findings in older women of associations between both LPA and MVPA and lower CRP.^42^ Our findings are important as they show that greater time spent in LPA in early old age, which makes up much of the time spent in PA by older adults,^43^ is associated with a better CVD biomarker profile. It also highlights marked sex differences, with associations being stronger in women and driven largely by sex differences in body composition.

### Explanation of Findings

Our findings suggest that 1 way through which PA may lower CVD risk is by improving physiological and biochemical functions of blood vessels, as indicated by associations of LPA and MVPA with inflammatory markers, adipokines, and t‐PA. The findings also suggest that increased sedentary time may be adversely related to endothelial function as indicated by its relation to these same markers, including to IL‐6, t‐PA, and leptin independently of MVPA. Their association with these biomarkers might mediate effects of PA and sedentary time on structural cardiovascular remodeling,^44^, ^45^ and these effects might be more apparent in those with vascular disease.^46^

That stronger associations were observed for women than men suggests that sex differences in response to PA and sedentary time may exist.^21^ For instance, it is thought that the modulation of the cardiovascular system by PA in older adults is in part affected by endogenous levels of sex hormones.^21^ Sex differences in a host of cardiovascular factors could contribute to the heterogeneous effects of PA on these CVD biomarkers.^21^ For example, these differences may be partially explained by differences in body composition responses to PA. This is supported by the fact that associations were not significantly different between men and women once differences in body composition were accounted for. This is further supported by findings from NSHD that MVPA in early old age was more strongly related to fat mass in women than men^27^ and by evidence of sex‐specific associations between testosterone and fat mass at age 60 to 64 years.^47^ Further, sex‐differences were generally less marked for MVPA than lower intensities, which suggests that higher PA intensities may be more important in men.^48^

Given that associations were only partly mediated by fat mass (albeit with greater mediation in women than men), adiposity may not be the only route through which PA benefits cardiovascular health,^49^ and this is consistent with studies showing persisting associations after adjustment for body size.^50^, ^51^ Further, our finding that fat mass mediated a large proportion of the associations between VO_2_max and CVD biomarkers is consistent with studies reporting that effects of cardiorespiratory fitness on cardiovascular structure^52^ and components of the metabolic syndrome^53^ were explained by adiposity. Finally, that PA was related to biomarkers after accounting for VO_2_max suggests that effects of PA on CVD may be at least partly independent of underlying fitness levels.^54^

### Methodological Considerations

An important strength of this study is the assessment of PA and sedentary time from combined heart rate and movement sensors to provide more precise estimates of activity intensity when compared with self‐reports. Other important strengths include the formal investigation of sex differences in these associations, investigating the mediating role of adiposity, an examination of how PA parameters relate a range of novel biomarkers each with differing underlying roles in CVD pathogenesis, and adjustment for important covariates. An important limitation of this study is that it was cross‐sectional, meaning we are unable to establish temporality. Reverse causation is possible and could partially explain our findings; this is particularly relevant for the observed PA and fitness differences between people with and without prevalent CVD. However, our main findings of association were broadly similar after exclusions in sensitivity analyses of prevalent disease, suggesting that associations did not differ substantially in those further along the disease trajectory. Additionally, our estimates of sedentary time included sleep and thus do not distinguish between sedentary time because of sleeping and sedentary time during waking hours. The weaker associations with CRP, IL‐6, t‐PA, and adiponectin when using only the (uniaxial) trunk acceleration may be because of PAEE obtained from combined heart rate and acceleration being better suited to capturing activities that affect these biomarkers. Also, participants who completed at least 4 minutes of the step test and therefore had an estimated VO_2_max were healthier than the larger sample with PA measurements and thus findings for VO2max may be less generalizable. Finally, doctor diagnoses were self‐reported and so could be prone to bias; however, self‐reported diabetes mellitus was validated against medical records in NSHD^55^ and self‐reported angina was found to correlate well with medical records in a similar‐aged sample of British men.^56^

### Implications and Conclusions

This study showed that in early old age, an important time of transition between work and retirement when behavior change may be possible,^57^ higher PAEE and greater time spent in LPA and MVPA and less sedentary time were related to more favorable CVD biomarker profiles. The associations found were stronger in women than men and remained after adjustment for covariates. Fat mass only partially mediated the main associations, although it was an important mediator, particularly in women. That is, the greater fat mass of women for a given body mass index explained why associations were stronger among women than men. Further, as sedentary time and LPA were both related to inflammatory and endothelial markers and adipokines independently of MVPA, our findings suggest that it is important for sedentary and inactive older adults to be supported to replace time spent sedentary with any intensity of PA.

## Author Contributions

Elhakeem, Cooper, and Hardy designed the study. Elhakeem performed statistical analysis and produced the first article draft. Elhakeem, Cooper, Whincup, Brage, Kuh, and Hardy contributed to development of the draft and read and approved its final version.

## Sources of Funding

The MRC NSHD, Cooper, Kuh, and Hardy are supported by the UK Medical Research Council (MC_UU_12019/1, MC_UU_12019/4, and G1001143). Brage is also supported by the UK Medical Research Council (MC_UU_12015/3). The funder had no direct involvement in this study.

## Disclosures

None.

## Supporting information


**Table S1.** Methods and Interassay Coefficients of Variation (CV) for Biomarkers Assessed From Blood Samples at Age 60 to 64 Years
**Table S2.** Mean Percentage Difference (95% Confidence Intervals) in Biomarkers Per Standard Deviation Increases in Time Spent Sedentary (SED), in Light‐ (LPA) and Moderate‐to‐Vigorous‐Intensity Physical Activity (MVPA), and Overall Physical Activity Energy Expenditure (PAEE) at Age 60 to 64: After Exclusion of Participants With Doctor‐Diagnosed Cardiovascular Disease
