# Supplementary material for: Physical Activity, Sedentary Time, and Cardiovascular Disease Biomarkers at Age 60 to 64 Years
Source: J Am Heart Assoc. 2018 Aug 8;7(16):e007459. doi: 10.1161/JAHA.117.007459 (PMC6201385; doi:10.1161/JAHA.117.007459)
Supplement: Supplementary file 1 — Table S1. Methods and Interassay Coefficients of Variation (CV) for Biomarkers Assessed From Blood Samples at Age 60 to 64 Years Table S2. Mean Percentage Difference (95% Confidence Intervals) in Biomarkers Per Standard Deviation Increases in Time Spent Sedentary (SED), in Light‐ (LPA) and Moderate‐to‐Vigorous‐Intensity Physical Activity (MVPA), and Overall Physical Activity Energy Expenditure (PAEE) at Age 60 to 64: After Exclusion of Participants With Doctor‐Diagnosed Cardiovascular Disease [file JAH3-7-e007459-s001.pdf]

SUPPLEMENTAL MATERIAL

Table S1. Methods and interassay coefficients (CV) of variation for biomarkers assessed from blood samples at age 60-64 years.

Cardiovascular disease biomarker	Units	Assay/Method	CV (%)
E-selectin	ng/ml	High sensitivity ELISA	<10.0%
C-reactive protein (CRP)	mg/l	Particle-enhanced immunoturbidimetric assay	4.3% at 3.4 mg/L<br>1.8% at 11.9 mg/L
Interleukin-6 (IL6)	pg/ml	Enzyme-linked immunosorbent assay (ELISA)	6.5%
Tissue plasminogen activator (tPA)	ng/ml	ELISA	6.6%
Leptin	ng/ml	In-house radioimmunoassay validated against commercially available assays	<10.0%
Adiponectin	μg/ml	ELISA	<7.5%

*Any results that fell below the lower detection limit of the assay were assigned a notional value, obtained by dividing the lower detection limit by the square root of 2.

Table S2. Mean percentage difference (95% confidence intervals) in biomarkers per standard deviation increases in time spent sedentary (SED), in light (LPA) and moderate-to-vigorous intensity physical activity (MVPA), and overall physical activity energy expenditure (PAEE) at age 60-64: after exclusion of participants with doctor-diagnosed cardiovascular disease.

	Men			Women		
	Model 1	Model 2	Model 3	Model 1	Model 2	Model 3
CRP (♂=684; ♀=756)						
SED	7.2 (0.5, 13.9)	4.7 (-1.9, 11.3)	5.4 (-1.2, 12.0)	17.2 (11.0, 23.4)	15.6 (9.2, 22.0)	8.8 (24.2, 15.1)
LPA	-4.8 (-11.6, 1.9)	-4.6 (-11.3, 2.1)	-4.0 (-10.5, 2.6)	-14.2 (-20.5, -8.0)	-13.1 (-19.5, -6.6)	-6.8 (-13.1, -0.5)
MVPA	-8.7 (-15.2, -2.2)	-6.1 (-12.6, 0.4)	-3.8 (-10.3, 2.7)	-17.9 (-23.9, -11.8)	-14.7 (-20.8, -8.5)	-9.2 (-15.2, -3.2)
PAEE	-9.6 (-16.2, -3.1)	-7.7 (-14.3, -1.1)	-5.5 (-12.1, 1.1)	-19.9 (-26.1, -13.7)	-17.5 (-23.9, -11.1)	-10.5 (-16.9, -4.1)
IL-6 (♂=678; ♀=751)						
SED	7.7 (2.4, 13.1)	6.0 (0.7, 11.4)	5.0 (-0.03, 10.3)	16.3 (11.2, 21.4)	14.3 (9.0, 19.5)	10.1 (4.8, 15.4)
LPA	-4.4 (-9.7, 1.0)	-3.5 (-8.9, 1.8)	-3.1 (-8.3, 2.2)	-13.7 (-18.8, -8.9)	-12.1 (-17.3, -6.8)	-8.2 (-13.5, -2.9)

MVPA	-11.0 (-16.1, -5.9)	-8.2 (-13.3, -3.1)	-6.6 (-11.7, -1.5)	-16.2 (-21.1, -11.3)	-13.1 (-18.1, -8.1)	-9.6 (-14.6, -4.6)
PAEE	-11.1 (-16.3, -6.0)	-8.9 (-14.1, -3.6)	-7.3 (-12.5, -2.1)	-18.1 (-23.2, -13.1)	-15.6 (-20.8, -10.3)	-11.3 (-16.6, -5.9)
t-PA (♂=623; ♀=681)						
SED	11.2 (6.2, 16.1)	9.9 (4.8, 15.0)	8.5 (3.6, 13.5)	11.7 (7.0, 16.5)	10.8 (5.7, 15.8)	6.2 (1.2, 11.3)
LPA	-10.9 (-15.8, -5.9)	-10.1 (15.1, -5.0)	-9.2 (-14.1, -4.4)	-10.2 (-15.0, -5.4)	-9.0 (-14.0, -3.9)	-5.0 (-10.0, -0.04)
MVPA	-6.1 (-10.9, -1.3)	-4.1 (-9.0, 0.8)	-2.1 (-6.9, 2.7)	-10.3 (-14.9, -5.8)	-9.9 (-14.7, -5.2)	-5.9 (-10.6, -1.2)
PAEE	-10.3 (-15.1, -5.4)	-8.6 (-13.6, -3.6)	-6.6 (-11.5, -1.6)	-12.1 (-16.8, -7.4)	-11.5 (-16.5, -6.5)	-6.7 (-11.7, -1.6)
E-selectin (♂=678; ♀=752)						
SED	1.7 (-1.6, 5.0)	0.9 (-2.4, 4.2)	0.2 (-3.1, 3.5)	2.4 (-0.8,5.6)	2.5 (-0.9, 5.9)	0.5 (-3.0, 4.0)
LPA	-1.3 (-4.6, 2.0)	-1.0 (-4.3, 2.3)	-0.6 (-3.9, 2.6)	-1.8 (-5.1, 1.4)	-1.8 (-5.2, 1.6)	-0.09 (-3.5, 3.4)
MVPA	-1.8 (-5., 1.4)	-0.2 (-3.5, 3.0)	0.9 (-2.3, 4.1)	-3.0 (-6.1, 0.2)	-3.0 (-6.2, 0.2)	-1.5 (-4.8, 1.8)
PAEE	-2.2 (-5.4, 1.0)	-0.8 (-4.1, 2.5)	0.3 (-2.9, 3.6)	-2.8 (-6.0, 0.4)	-2.9 (-6.3, 0.5)	-0.9 *-4.4, 2.6)
Leptin (♂=680; ♀=753)						
SED	10.8 (5.2, 16.3)	7.6 (2.1, 13.0)	3.4 (-0.7, 7.6)	23.2 (17.8, 28.7))	18.8 (13.2, 24.3)	5.3 (1.0, 9.6)
LPA	-5.8 (-11.4, -0.2)	-3.3 (-8.7, 2.1)	-1.2 (-5.3, 2.9)	-19.8 (-25.3, -14.3)	-15.7 (-21.3, -10.1)	-3.7 (-7.9, 0.6)
MVPA	-16.0 (-21.3, -10.7)	-12.8 (-17.9, -7.6)	-6.5 (-10.4, -2.5)	-22.4 (-27.7, -17.1)	-17.8 (-23.0, -12.5)	-6.9 (-10.9, -3.0)

PAEE	-16.3 (-21.7, -10.9)	-12.7 (-18.0, -7.4)	-6.3 (-10.3, -2.2)	-25.7 (-31.1, -20.3)	-20.7 (-26.2, -15.2)	-6.8 (-11.0, -25.0)
Adiponectin (♂=680; ♀=752)						
SED	-6.7 (-11.7, -1.8)	-4.9 -10.1, 0.3)	-4.0 (-9.1, 1.1)	-11.4 (-15.6, -7.2)	-8.7 (-13.1, -4.3)	-5.2 (-9.6, -0.8)
LPA	6.0 (1.0, 11.0)	4.4 (-0.7, 9.6)	4.0 (-1.0, 9.1)	-11.3 (7.0, 15.5)	8.9 (4.5, 13.2)	5.7 (1.4, 10.1)
MVPA	5.0 (0.1, 9.8)	3. (-1.7, 8.2)	1.8 (-3.2, 6.7)	6.4 (2.3, 10.5)	3.6 (-0.6, 7.8)	0.6 (-3.6, 4.7)
PAEE	6.8 (1.9, 11.7)	4.9 (-0.2, 9.9)	3.4 (-1.7, 8.4)	10.2 (6.0, 14.5)	7.3 (2.9, 11.6)	3.5 (-0.9, 7.9)

Model 1: adjusted for age. Model 2: adjusted for age, SEP, smoking history, long-term illness, health problem or disability, blood pressure and medication use. Model 3: as for model 2 plus added adjustment for fat mass index.
